# Heterogeneous response and progression patterns reveal phenotypic heterogeneity of tyrosine kinase inhibitor response in metastatic renal cell carcinoma

**DOI:** 10.1186/s12916-016-0729-9

**Published:** 2016-11-14

**Authors:** Shanthini M. Crusz, Yen Zhi Tang, Shah-Jalal Sarker, Warner Prevoo, Irfan Kiyani, Luis Beltran, John Peters, Anju Sahdev, Axel Bex, Thomas Powles, Marco Gerlinger

**Affiliations:** 1 Barts Cancer Institute, Queen Mary University of London, London, UK; 2 Department of Radiology, St Bartholomews Hospital, London, UK; 3 Departments of Surgical and Medical Oncology, The Netherlands Cancer Institute, Amsterdam, The Netherlands; 4 Institute of Nuclear Medicine, University College Hospital, London, UK; 5 Department of Surgery, Whipps Cross Hospital, London, UK; 6 Centre for Evolution and Cancer, The Institute of Cancer Research, 237 Fulham Road, London, SW3 6JB UK; 7 The Royal Marsden Hospital, London, UK

**Keywords:** Anti-angiogenic treatment, Drug resistance, Intratumour heterogeneity, Kidney cancer, RECIST

## Abstract

**Background:**

Molecular intratumour heterogeneity (ITH) is common in clear cell renal carcinomas (ccRCCs). However, it remains unknown whether this is mirrored by heterogeneity of drug responses between metastases in the same patient.

**Methods:**

We performed a retrospective central radiological analysis of patients with treatment-naïve metastatic ccRCC receiving anti-angiogenic tyrosine kinase inhibitors (TKIs) (sunitinib or pazopanib) within three similar phase II trials. Treatment was briefly interrupted for cytoreductive nephrectomy. All patients had multiple metastases that were measured by regular computed tomography scans from baseline until Response Evaluation Criteria In Solid Tumours (RECIST)-defined progression. Each metastasis was categorised as responding, stable or progressing. Patients were classed as having a homogeneous response if all lesions were of the same response category and a heterogeneous response if they differed.

**Results:**

A total of 115 metastases were assessed longitudinally in 27 patients. Of these patients, 56% had a heterogeneous response. Progression occurred through the appearance of new metastases in 67%, through progression of existing lesions in 11% and by both in 22% of patients. Despite RECIST-defined progression, 57% of existing metastases remained controlled. The sum of controlled lesions was greater than that of uncontrolled lesions in 47% of patients who progressed only with measurable new lesions.

**Conclusions:**

We identified frequent ITH of anti-angiogenic TKI responses, with subsets of metastases responding and progressing within individual patients. This mirrors molecular ITH and may indicate that anti-angiogenic drug resistance is confined to subclones and not encoded on the trunk of the tumours’ phylogenetic trees. This is clinically important, as patients with small-volume progression may benefit from drug continuation. Predominant progression with new rather than in existing metastases supports a change in disease biology through anti-angiogenics. The results highlight limitations of RECIST in heterogeneous cancers, which may influence clinical trial data validity. This analysis requires prospective confirmation.

**Trial registration:**

European Clinical Trials Database(EudraCT): 2009-016675-29, registered 17 March 2010; EudraCT: 2006-004511-21, registered 09 March 2007; EudraCT: 2006-006491-38, registered 22 December 2006.

**Electronic supplementary material:**

The online version of this article (doi:10.1186/s12916-016-0729-9) contains supplementary material, which is available to authorized users.

## Background

Extensive genetic, transcriptomic, signalling pathway activity and predictive and prognostic biomarker heterogeneity have been shown within and between clear cell renal carcinoma (ccRCC) primary tumours and metastases [1–4]. Yet, it has not been investigated whether this molecular intratumour heterogeneity (ITH) is mirrored by heterogeneous response and progression patterns of different metastases within individual patients during anti-angiogenic tyrosine kinase inhibitor (TKI) treatment, which is standard of care in the first- and second-line treatment of metastatic ccRCCs [5–7]. This information is clinically relevant, as uniform responses of multiple metastatic sites would suggest that drug sensitivity or resistance is determined by a common molecular characteristic encoded on the trunk of the tumour’s phylogenetic tree [8]. In contrast, frequent occurrence of heterogeneity may hinder development of predictive biomarkers to identify patients likely to benefit from these treatments. Heterogeneous progression patterns may also complicate clinical decisions. If small-volume progression is common despite ongoing control of the disease bulk, systemic therapy continuation or local treatment modalities could be offered and biopsy approaches to identify drug-resistant subclones could be informative, whereas progression in all or most metastases may require a switch to a different class of systemic treatment.

## Methods

### Patients and treatment

Patients with treatment-naïve metastatic ccRCC enrolled in three similar single-arm phase II studies of first-line pazopanib (study A: PANTHER) or sunitinib (study B: SuMR, study C: N06SUN) [9–12] and interval nephrectomy were eligible for inclusion into this post hoc radiological substudy. All trials were approved by an ethics committee and entered in a clinical trials register (A: EudraCT 2009-016675-29, B: EudraCT 2006-004511-21, C: EudraCT 2006-006491-38). All patients provided signed written informed consent. Sunitinib (50 mg PO once daily for 4 weeks, 2 weeks off drug) was administered for two (study C) or three cycles (study B) and pazopanib (800 mg PO once daily) was administered for 12–16 weeks prior to planned cytoreductive nephrectomy. Drug therapy was restarted following recovery from surgery until radiological disease progression.

**Fig. 1:**
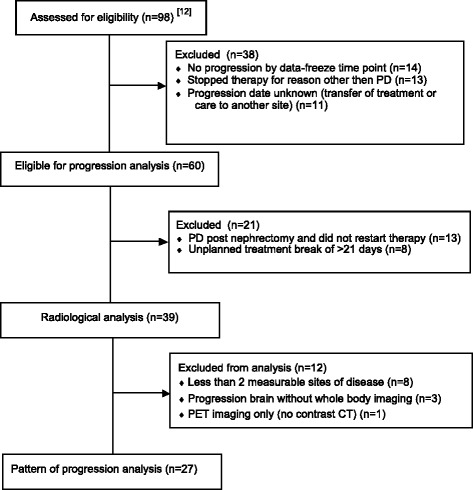
Flowchart of patient selection for radiological heterogeneity analysis. *PD* progressive disease

Data were available from 98 patients included in a prior interim analysis of these three trials [12]. To assess progression patterns, only patients with Response Evaluation Criteria In Solid Tumours (RECIST) 1.1-defined disease progression by the data-freeze time point (01 May 2014) were eligible for assessment (*n* = 60) (Fig. 1). Patients who underwent nephrectomy were required to have restarted the drug after surgery to be included for analysis. For six patients who restarted treatment but had progressed during the planned drug-free interval peri-nephrectomy, the post-nephrectomy scan was used as baseline to avoid biases that may result from interval progression. Further, patients who had unplanned treatment breaks >21 days were excluded (*n* = 8), as interval progression was likely in those cases. These criteria resulted in 27 patients being eligible for assessment in the final analysis of whole body computed tomography (CT) or positron emission tomography (PET)-CT imaging of two or more measurable lesions.

### Disease assessment

CT or PET-CT scans of at least the chest and abdomen were performed according to trial protocols at baseline, before and after surgery, and at 3- month intervals thereafter until disease progression or if clinically indicated. CT scans or CT components of PET-CT scans were re-analysed centrally within the lead centre of each trial by two radiologists (studies A/B: YT, study C: WP) according to modified RECIST 1.1 criteria. The primary renal lesion was excluded from the analysis as this was removed surgically in all patients except 11, who did not undergo surgery due to progression before scheduled nephrectomy or patient choice. All measurable metastases at baseline were included for response pattern assessments (i.e. more than five lesions in total), including lung nodules between 5–10 mm if unequivocally considered metastatic. Unidimensional measurements were performed to the nearest millimetre with picture archiving and communication system (PACS) software on all scans from baseline to progression.

### Statistical analysis

A Fisher’s exact test was used to test the association between lesion response category and lesion size and between progression pattern and treatment type. A proportion test was used to compare the heterogeneous response type for pazopanib- and sunitinib-treated patients. Intercooled Stata 13 (Stata Corporation, College Station, TX, USA) was used for the statistical analysis. A *p* value of less than 0.05 was considered significant.

## Results

### Patients and radiological assessment

Ninety-eight patients with metastatic ccRCC scheduled for cytoreductive nephrectomy were included into one of three similar phase II trials of peri-operative therapy with sunitinib or pazopanib. Twenty-seven patients fulfilled the inclusion criteria for this radiological subanalysis (Fig. 1). Characteristics of this subgroup were similar to those of all patients included into the trials (Table 1), indicating that the selection criteria for this subanalysis did not introduce major known biases.

**Table 1:** Patient characteristics

Characteristic		Overall cohort [12]	Heterogeneity study
Number of patients		98	27
Male patients (*n*, %)		75 (77%)	23 (85%)
Median age (years)		59 (range 37–78)	59 (range 34–73)
MSK risk group	Intermediate	70 (71%)	18 (67%)
	Poor	28 (29%)	9 (33%)
Nephrectomy	Yes	62 (63%)	16 (59%)
Organ sites affected by metastases	1	30 (31%)	8 (30%)
	2	39 (40%)	13 (48%)
	3+	29 (29%)	6 (22%)

*MSK* Memorial Sloan Kettering [22]

Characteristics of all 98 patients included into the three phase II trials compared to those of the 27 patients in the radiological heterogeneity substudy

All measurable lesions were followed on regular CT scans until RECIST-defined progression (Additional file 1: Table S1). In order to assess intraobserver variability, 20% of all patients (*n* = 5) were chosen randomly for a re-analysis by the same radiologist blinded to previous measurements. The Pearson correlation coefficient of 0.99 indicated highly reproducible measurements.

### Heterogeneity at best response

**Fig. 2:**
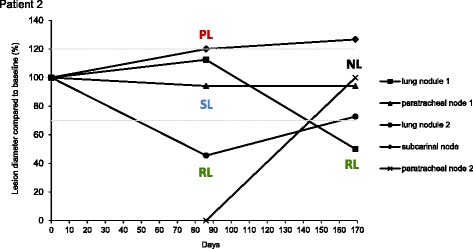
Example of individual lesion response assessments within one patient. Lesion size on each CT scan relative to the size on the baseline scan was calculated until RECIST-defined progression. Based on the best response that was achieved over the treatment period, each lesion was categorised either as a Responding Lesion (*RL*, 30% or greater decrease in diameter compared to baseline), Progressing Lesion (*PL*, 20% or greater increase in diameter compared to baseline) or Stable Lesion (*SL*, all remaining lesions). The emergence of new lesions (*NL*) was also recorded

We first assessed whether multiple metastatic sites within individual patients responded similarly to the drug or whether heterogeneous radiological responses occurred. Larger lesions may take longer to respond than smaller lesions; thus, each metastasis was categorised based on the best response achieved over the treatment period to mitigate the impact of such response dynamics. We identified the minimal diameter of each of 115 measurable metastases during drug therapy and compared it to the diameter of the same lesion at baseline (Additional file 1: Table S1). Based on the relative size change, each metastasis was classified into one of three RECIST-analogous response categories: Responding Lesions (RLs) decreased in size by 30% or more compared to baseline, Progressing Lesions (PLs) increased in size by 20% or more and all other lesions were classified as Stable Lesions (SLs) (example in Fig. 2; Additional file 2: Figure S1).

**Fig. 3:**
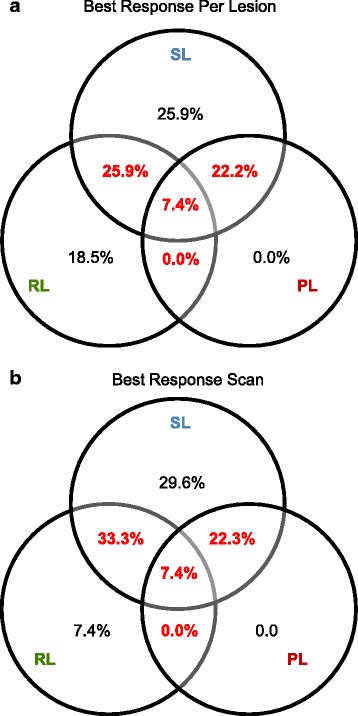
Venn diagram of response patterns. Percentage of 27 patients with the indicated combination of lesion response categories based on **a** the assessment of the best response achieved per lesion and **b** assessment on the specific scan showing the best overall response. *RL* Responding Lesion, *SL* Stable Lesion, *PL* Progressing Lesion

Each patient was assigned either to the group with homogeneous drug responses (all lesions within the same response category) or heterogeneous responses (lesions in at least two of the three response categories). Fifteen patients (55.6%) showed a heterogeneous response and 12 patients (44.4%) a homogeneous response (Fig. 3, Additional file 3: Table S2). In 8 patients (29.6%), at least one metastasis showed outright progression (PL), while others were stable or responded. Heterogeneous responses were more frequent in patients treated with pazopanib (7/8 = 88%) than sunitinib (8/19 = 44%) (*p* = 0.03), but did not differ in patients who had dose reductions due to toxicities (5/9 = 56%) compared to those who did not (10/18 = 56%). The response patterns were similar and there was no statistically significant difference for patients who underwent nephrectomy (8/16 = 50% heterogeneous responses) and those who did not have a nephrectomy (7/11 = 64% heterogeneous responses, *p* = 0.70). Thus, heterogeneous responses cannot be explained by suboptimal dosing or nephrectomy.

In addition to the analysis based on best response per lesion, we also evaluated response heterogeneity by comparing lesion sizes at baseline with the scan showing the best overall response to treatment (lowest observed sum of all measurable lesion diameters). This approach, which is more similar to radiological practice, found heterogeneous responses in a similar fraction of patients (63%) (Fig. 3).

**Fig. 4:**
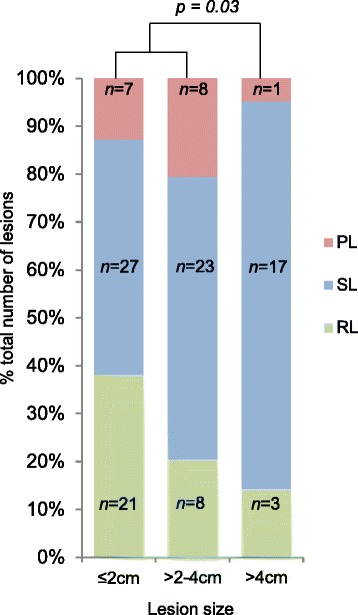
Best achieved response by baseline lesion size. Best response achieved by each individual lesion compared to its size at baseline. *p* value refers to SLs compared to RLs and PLs in ≤4 cm lesions versus >4 cm lesions. *RL* Responding Lesion, *SL* Stable Lesion, *PL* Progressing Lesion

Metastases were catergorised as small (≤2 cm, *n* = 55), intermediate (>2–4 cm, *n* = 39) and large lesions (>4 cm, *n* = 21) to assess how the size on baseline scan relates to the best response achieved. Large lesions were significantly more stable compared to smaller and intermediate lesions together (*p* = 0.03) (Fig. 4). This may result from large fibrotic or necrotic components that may not change during therapy. Alternatively, the change of tumour volume which is necessary before a 20% increase or 30% decrease in diameter is detected may not be achievable for many large lesions within the treatment period [13]. However, 82% (94/115) of metastases were of small or intermediate size. After removing lesions measuring >4 cm from the analysis, 57% (13/23) of patients with at least two measurable metastases remaining still showed a heterogeneous response. Thus, the presence of large lesions is not the main driver of response heterogeneity.

This analysis demonstrates that heterogeneous responses with lesions in two or three different response categories are common in ccRCC. Thus, individual lesions can differ with respect to drug sensitivity, suggesting the molecular determinants of drug response are unlikely to be encoded on the trunk of these tumours’ phylogenetic trees.

### Heterogeneity at progression

**Fig. 5:**
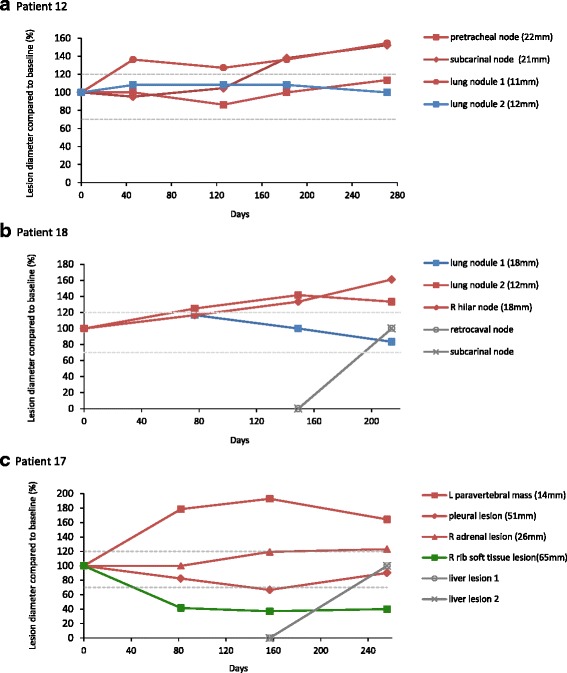
Patterns at progression. Examples of RECIST progression patterns. **a** ≥20% increase in size of existing disease from nadir defining progression. **b** New lesions and ≥20% increase in size of existing disease from nadir defining progression. **c** New lesions only defining progressive disease (*R* right, *L* left, *green line* responding lesion at progression, *blue line* stable lesion at progression, *red line* progressing lesion at progression, measurements as per size at baseline)

**Fig. 6:**
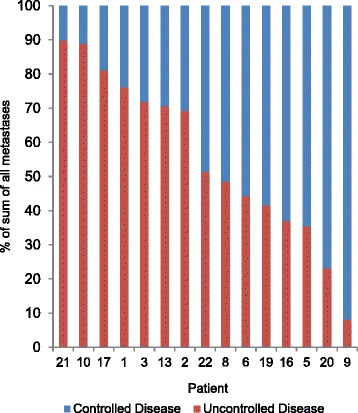
Controlled versus uncontrolled lesions in patients progressing with measurable new lesions only. The sum of diameters of controlled lesions (responding and stable lesions combined) and the sum of diameters of uncontrolled lesions (progressing and new lesions combined) is shown relative to the sum of all lesion diameters for 15 patients in whom only measurable new lesions defined progression

The analysis of drug resistance heterogeneity at RECIST-defined cancer progression was the next aim. Each metastasis was categorised as described into RL/SL/PL based on the diameter at progression compared to baseline; however, lesions that increased ≥20% compared to nadir were also labelled PL (Additional file 1: Table S1 and Additional file 4: Figure S2). Measurable and non-measurable new lesions (NLs) were also recorded. Only 3/27 patients (11%) had progression solely based on an increase in the sum of target lesions of ≥20% (example in Fig. 5a). Six patients (22%) showed progression through NL and a simultaneous increase in the sum of target lesions ≥20% (example in Fig. 5b), whereas NL alone defined progression in the remaining 18 patients (67%) (example in Fig. 5c). In 7 of these 18 patients, all 21 metastases that had already been present on the baseline scan were still classed as SL or RL at progression. No statistically significant difference in progression pattern was seen between patients treated with pazopanib compared to sunitinib (*p* = 0.68). As per RECIST criteria, the occurrence of any new malignant lesion defines progression, irrespective of its absolute size or the relative size in comparison to that of target lesions present from baseline. Thus, in cancers in which progression is predominantly driven by the occurrence of new lesions, RECIST criteria may frequently lead to treatment discontinuation while the bulk of the disease remains controlled. Indeed, the sum of controlled lesions (RLs and SLs) was greater than that of uncontrolled lesions (PLs and NLs) in 7/15 patients (47%) who progressed only with measurable new lesions (Fig. 6). Overall, 66/115 baseline metastases (57%) assessed at progression remained stable or were still responding at RECIST-defined progression, further supporting this notion. This may be consistent with the evolution of drug-resistant clones in a subset of metastases, whereas the majority of metastases may remain drug-sensitive.

## Discussion

This small study of 27 out of 98 patients who fulfilled the criteria for a radiological re-analysis demonstrates frequent anti-angiogenic drug response heterogeneity between ccRCC metastases both during early treatment and at progression. Due to the risks and technical difficulties of biopsying multiple metastatic sites, tissues for correlative molecular analyses were not available. However, the observed phenotypic heterogeneity during drug therapy resembles the genotypic and transcriptomic heterogeneity previously described in ccRCC [1–4]. Hence, it is conceivable that molecular alterations that determine treatment responses are unlikely to be encoded by early mutations, called truncal mutations, and that subclonal heterogeneity is a key driver of this intrapatient response heterogeneity. The variable drug responses of different ccRCC metastases further indicate that single biopsies will most likely be insufficient to identify patients who will progress early. Circulating tumour DNA sequencing, which may sample multiple metastatic sites simultaneously through the perfusing blood [14], or functional imaging that can detect individual drug-resistant metastases [15, 16] may be more suitable to identify such individuals.

Regardless of the underlying molecular mechanisms, our finding that drug sensitivity phenotypes often differ between metastases within patients adds to the challenges arising from ITH for precision cancer medicine [17]. Radiological response assessments based on RECIST criteria are commonly applied for treatment decision making in metastatic cancers. Yet, the impact of ITH on reliable determination of decisions to discontinue or switch to alternative therapy has not been thoroughly assessed. A pertinent finding was the large proportion of patients in whom a relatively small fraction of the entire disease bulk progressed whereas the remainder remained controlled. As pazopanib/sunitinib treatment was stopped at radiological progression, we could not investigate whether further metastases would start to progress soon if treatment had continued beyond progression. Nevertheless, this raises the possibility that pazopanib/sunitinib continuation, potentially in combination with focal therapies for small-volume drug-resistant disease, may lead to better outcomes than immediate stopping or switching to second-line therapy. To date, robust radiological tools and criteria to assess ITH have not been defined. Imaging approaches which can assess the fraction of the cancer load that is progressing within a patient, for example, through volumetric analysis, may be desirable to guide such decisions. Novel imaging approaches, such as CT texture analysis, functional MRI and other functional imaging modalities, could further assist in the detection of metastases that differ in their biological characteristics. Some of these technologies have already been shown to correlate with outcomes in cancer patients, including those with ccRCC [18].

The large proportion of cancers that progressed through new lesions alone (67%) may also influence clinical trial data interpretation and validity. The following example illustrates this: a small new lesion defines progression based on RECIST criteria even if several large lesions remain controlled. If such a patient enters a subsequent clinical trial using RECIST criteria for progression-free survival (PFS) assessment, the new lesion will be counted towards the diameter of all target lesions. Assuming the patient is treated with a drug with identical or similar efficacy and mechanism of action as used first line, the bulk of the cancer which has remained sensitive throughout will be controlled again and the contribution of the small drug-resistant lesion to the overall target lesion diameter is small. Hence, even if the new drug has identical activity to the one used during first-line therapy, the patient is likely to achieve a substantial increase in PFS compared to that achieved without treatment or with a less active drug. This highlights a potential limitation of RECIST in cancer types progressing predominantly with new lesions, and this is particularly relevant in ccRCC where anti-angiogenic agents with similar mechanisms have been assessed sequentially. For example, the phase III AXIS trial that reported a PFS gain from axitinib compared to the less potent sorafenib may be affected by such effects [19, 20]. The analysis of progression patterns when these patients failed first-line therapy could shed light on this subject. Whether similar benefit would be achieved by continuation of first-line anti-angiogenics is an important question.

Previous analyses of progression patterns in patients with metastatic ccRCC receiving the anti-angiogenic drugs bevacizumab or sorafenib found that new metastases alone and new metastases combined with an increase of existing disease defined disease progression in 18% and 10% of patients, respectively [21]. The higher proportion of new lesions in our study may reflect differences in tumour characteristics between the studies. All patients in our trials had presented with synchronous metastases, a feature of more aggressive ccRCCs [22]. Alternatively, the increased anti-angiogenic potency of sunitinib and pazopanib compared to sorafenib/bevacizumab may alter dissemination and recurrence patterns [23]. This has previously been suggested by studies showing anti-angiogenic agents to promote cancer invasiveness and dissemination in mouse models [24, 25]. A subsequent post hoc analysis of the phase III trial comparing sunitinib with interferon-α concluded that sunitinib did not alter tumour biology [26]. However, this analysis did not specifically assess the overall patterns of disease progression or the occurrence of new metastatic sites. Thus, although a survival benefit of targeted therapy has clearly been shown [27], these results warrant further investigation into the impact of anti-angiogenic TKIs on the biology and evolution of metastatic ccRCC.

Our study demonstrated heterogeneity of response and progression patterns on anti-angiogenic therapy. However, it is limited by the design of the phase II trials analysed. These were enriched for high-risk patients, and 16 patients underwent an interval nephrectomy which required a brief peri-operative treatment interruption (median 35 days, range 18–71 days). Based on the aims of this study, the analysis had to be restricted to patients with two or more assessable metastatic lesions who had progressed during ongoing anti-angiogenic therapy. Only 27 patients met these criteria and were included into the final analysis (Fig. 1). This small patient number could create potential biases, and the original studies were not powered for this retrospective analysis. Thus, our findings need confirmation in ideally prospective analyses of larger patient numbers to confirm results, and suitable data should be collected routinely in the context of large registration trials.

## Conclusions

Our results demonstrate phenotypic heterogeneity of anti-angiogenic TKI responses and resistance in patients with metastatic ccRCC. We hypothesise that these findings may be driven by the molecular ITH previously demonstrated in ccRCC. Although future confirmation of these results is required, this study clearly outlines some of the challenges arising from ITH for clinical trial interpretation and for clinical decision making. Incorporating response and resistance heterogeneity assessments in clinical practice may increase patient benefit in the future.

## Additional files

Additional file 1: Table S1. Patient details and lesion measurements: lesion measurement for all scans included in analysis. (XLSX 57 kb)
Additional file 2: Figure S1. Response heterogeneity. All patient lesions as percentage change in diameter on CT scan relative to size at baseline until progressive disease. (PDF 52 kb)
Additional file 3: Table S2. Summary of patient response and progression classification. (DOCX 22 kb)
Additional file 4: Figure S2. Patient graphs. Percentage change in diameter on CT scan relative to size at baseline until RECIST-defined progressive disease. (PDF 195 kb)

## Abbreviations

ccRCC: Clear cell renal carcinoma
ITH: Intratumour heterogeneity
PFS: Progression-free survival
PL: Progressing Lesion
RECIST: Response Evaluation Criteria In Solid Tumours
RL: Responding Lesion
SL: Stable Lesion
TKI: Tyrosine kinase inhibitor
