# Supplementary material for: Heterogeneous response and progression patterns reveal phenotypic heterogeneity of tyrosine kinase inhibitor response in metastatic renal cell carcinoma
Source: BMC Med. 2016 Nov 14;14:185. doi: 10.1186/s12916-016-0729-9 (PMC5108081; doi:10.1186/s12916-016-0729-9)

Figure S1 – Response Heterogeneity. All patient lesions as percentage change in diameter on CT scan relative to size at baseline until progressive disease.
(R = right, L = left, P = patient, dash line = new lesion)

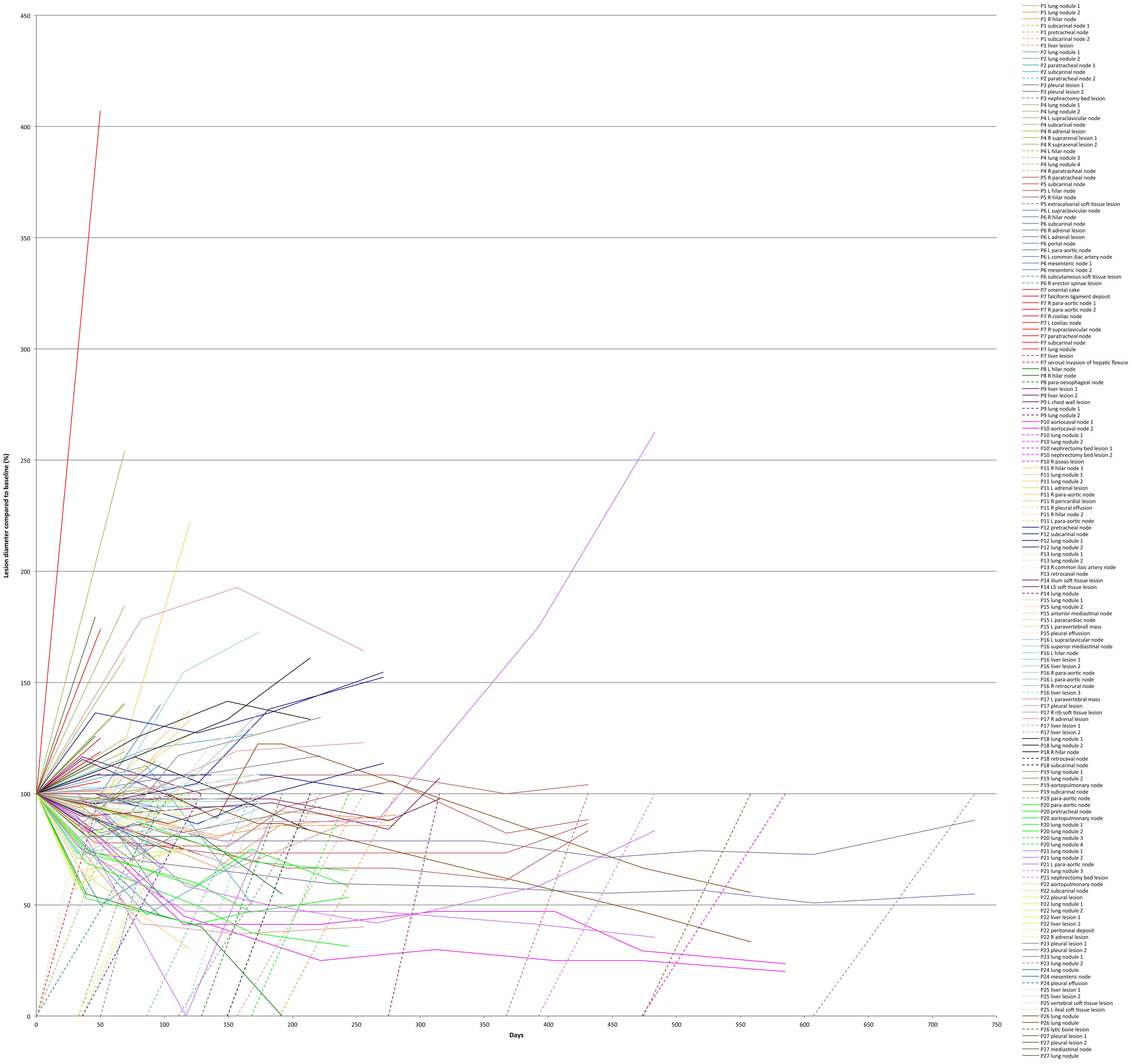

Supplement: Additional file 2: Figure S1. — Response heterogeneity. All patient lesions as percentage change in diameter on CT scan relative to size at baseline until progressive disease. (PDF 52 kb) [file 12916_2016_729_MOESM2_ESM.pdf]
