# Supplementary material for: Heterogeneous response and progression patterns reveal phenotypic heterogeneity of tyrosine kinase inhibitor response in metastatic renal cell carcinoma
Source: BMC Med. 2016 Nov 14;14:185. doi: 10.1186/s12916-016-0729-9 (PMC5108081; doi:10.1186/s12916-016-0729-9)
Supplement: Additional file 3: Table S2. — Summary of patient response and progression classification. (DOCX 22 kb) [file 12916_2016_729_MOESM3_ESM.docx]

**Table S2 – Summary of Patient Response and Progression Classification**

| **Patient Number** | **Drug** | **Response Category** | **Progression Category** | **NL with Controlled Pre-existing Disease at Progression*** | **Diameter Controlled Lesions (RL+SL) at Progression (mm)** | **Diameter Uncontrolled Lesions (PL+NL) at Progression (mm)**** |
| --- | --- | --- | --- | --- | --- | --- |
| 1 | pazopanib | heterogeneous | new lesions | No | 40 | 127 |
| 2 | pazopanib | heterogeneous | new lesions | No | 20 | 45 |
| 3 | pazopanib | heterogeneous | new lesions | No | 38 | 97 |
| 4 | pazopanib | heterogeneous | new lesions and ≥20% increasing in size of existing disease from nadir | No | 19 | 352 |
| 5 | pazopanib | heterogeneous | new lesions | No | 53 | 29 |
| 6 | pazopanib | heterogeneous | new lesions | No | 121 | 96 |
| 7 | pazopanib | heterogeneous | new lesions and ≥20% increasing in size of existing disease from nadir | No | 177 | 200 |
| 8 | pazopanib | homogeneous | new lesions | Yes | 17 | 16 |
| 9 | sunitinib | homogeneous | new lesions | Yes | 217 | 19 |
| 10 | sunitinib | homogeneous | new lesions | Yes | 8 | 64 |
| 11 | sunitinib | heterogeneous | new lesions and ≥20% increasing in size of existing disease from nadir | No | 21 | 224 |
| 12 | sunitinib | heterogeneous | ≥20% increasing in size of existing disease from nadir | N/A | 12 | 74 |
| 13 | sunitinib | heterogeneous | new lesions | No | 38 | 91 |
| 14 | sunitinib | homogeneous | new lesions and ≥20% increasing in size of existing disease from nadir | No | 40 | 112 |
| 15 | sunitinib | homogeneous | new lesions | Yes | 94 | N/A |
| 16 | sunitinib | homogeneous | new lesions | No | 229 | 135 |
| 17 | sunitinib | heterogeneous | new lesions | No | 26 | 112 |
| 18 | sunitinib | heterogeneous | new lesions and ≥20% increasing in size of existing disease from nadir | No | 15 | 83 |
| 19 | sunitinib | heterogeneous | new lesions | No | 77 | 55 |
| 20 | sunitinib | homogeneous | new lesions | Yes | 43 | 13 |
| 21 | sunitinib | homogeneous | new lesions | No | 6 | 53 |
| 22 | sunitinib | heterogeneous | new lesions | No | 119 | 126 |
| 23 | sunitinib | homogeneous | new lesions and ≥20% increasing in size of existing disease from nadir | No | 0 | 106 |
| 24 | sunitinib | homogeneous | new lesions | Yes | 88 | N/A |
| 25 | sunitinib | homogeneous | ≥20% increasing in size of existing disease from nadir | N/A | 26 | 103 |
| 26 | sunitinib | homogeneous | new lesions | Yes | 10 | N/A |
| 27 | sunitinib | heterogeneous | ≥20% increasing in size of existing disease from nadir | N/A | 109 | 104 |

*N/A: Patients with ≥20% increasing in size of existing disease from nadir therefore analysis not applicable. ** N/A: Patients who have progressed with non-measurable new lesions only therefore analysis not applicable. NL = new lesion, RL = responding lesion, SL – stable lesions, PL = progressing lesion.
