# Supplementary figures and images for: Heterogeneous response and progression patterns reveal phenotypic heterogeneity of tyrosine kinase inhibitor response in metastatic renal cell carcinoma
Source: BMC Med. 2016 Nov 14;14:185. doi: 10.1186/s12916-016-0729-9 (PMC5108081; doi:10.1186/s12916-016-0729-9)

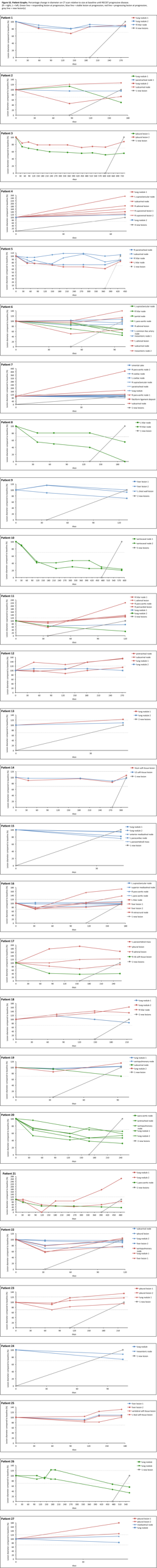

Supplement: Additional file 4: Figure S2. — Patient graphs. Percentage change in diameter on CT scan relative to size at baseline until RECIST-defined progressive disease. (PDF 195 kb) [file 12916_2016_729_MOESM4_ESM.pdf]
